# Impact of Silver Diamine Fluoride Therapy on the Oral Health-Related Quality of Life of Preschool Children with Behavioral Problems after Three Months: A Pilot Study

**DOI:** 10.3390/jcm11113071

**Published:** 2022-05-29

**Authors:** Sarra Altner, Daniel Stroj, Katrin Bekes

**Affiliations:** Department of Pediatric Dentistry, University Clinic of Dentistry, Medical University of Vienna, Sensengasse 2a, 1090 Vienna, Austria; sarra.altner@meduniwien.ac.at (S.A.); n1447331@students.meduniwien.ac.at (D.S.)

**Keywords:** silver diamine fluoride, oral health-related quality of life, pediatric dentistry, children, dental caries, parental satisfaction, behavioral problems

## Abstract

Background: In Austria, almost every second child has caries. The consequences of untreated carious lesions are infections, pain, and limitations in everyday life. The aim of this study was to evaluate the influence of silver diamine fluoride (SDF) treatment on the oral health-related quality of life (OHRQoL) of uncooperative children aged 0–5 years using the German version of the Early Childhood Oral Health Impact Scale (ECOHIS-G). Methods: This prospective study was conducted at the Department of Paediatric Dentistry at the Medical University of Vienna. Preschool children with behavioral problems and carious lesions that required SDF application were included. The ECOHIS-G questionnaire was given to the caregiver before (T0) and three months (T1) after treatment. Using descriptive analysis and the Wilcoxon Signed-Rank test, changes in the ECOHIS scores were evaluated and tested for significance. Results: A total of 30 children aged 0–5 years were enrolled and received SDF treatment. At baseline, the total ECOHIS score was 21.4 (±8.5). Three months after therapy, a significant improvement was achieved (16.3 [±5.6], *p* < 0.05). Significantly better scores were observed in six subdomains, especially in “child function” (3.9 [±2.0]) and “child symptoms” (2.0 [±1.3]) (*p* < 0.05). Conclusions: Treatment of carious lesions with SDF in the primary dentition resulted in an improvement in the OHRqoL of children with behavioral problems.

## 1. Introduction

Early childhood caries (ECC) is a disease that describes the presence of one or more decayed, missing, or filled primary teeth in children under the age of six [1]. It is still one of the main challenges in pediatric dentistry on a global level with a prevalence of up to 46% [2]. Even in developed countries, such as Austria, around 42% of children are affected [3]. If ECC remains untreated the body weight, growth, and school performance can be affected, which leads to a compromised oral health-related quality of life [4,5].

Treatment of ECC can be complicated due to the unforeseeable and changing compliance of a patient [6]. In particular, invasive treatments of caries lesions can exacerbate this underlying challenge. Tackling these situations often requires pharmacological and behavior management approaches, which include treatment with dental general anesthesia (DGA) or sedation [7,8]. However, not every patient fulfills the requirements for sedation, which is only justified in the case of severe and advanced dental decay [9]. Even those who are suitable may not undergo this treatment due to the high costs and the associated risks [10]. Particularly for patients younger than three, the potential neurological side effects that come with general anesthesia mean that this treatment should be regarded as a last resort, which needs to be duly justified [11]. If only a few teeth are affected, one ought to look for another solution. Silver diamine fluoride (SDF), first used in Japan in the 1970s, did not gain wider attention until a few years ago, when it was recognized as an alternative noninvasive treatment for children with behavioral problems [12]. It is a solution consisting of silver, ammonia, and fluoride that has proven to be efficient in directly inhibiting dentin and enamel caries, thereby delaying surgical intervention [13]. After treatment, SDF, due to its antibacterial function, often leads to direct pain relief and improved dental functioning, as it allows children with difficulties in eating and chewing to be pain free, thereby increasing their quality of life [14]. A possible drawback is associated with the oxidizing effects of SDF, which leads to an irreversible black staining of the carious lesions. This is often a hurdle for parents, who do not want their children to suffer aesthetic impairments [15].

It is these associated costs and benefits that leave an open question of whether aesthetic or dental function aspects predominate with regard to the overall quality of life and thus whether SDF is a viable solution in societies in which the aesthetic aspects of dental treatment play an important role.

Over the past several years, the concept of oral health-related quality of life (OHRQoL) has attained importance in the field of dentistry [16]. The Early Childhood Oral Health Impact Scale (ECOHIS) is a questionnaire designed for adult caregivers that assesses the impact of oral health problems and related treatment experiences on the quality of life of preschool children and their families [17,18]. It is an ideal instrument to assess the relative weight of both improved dental functioning due to treatment with SDF and possible aesthetic limitations. It does this by not only considering the perspective of the child but also by including the caregiver’s perception with regard to the child’s wellbeing.

In a recently conducted meta-analysis comparing the influence of SDF on the OHRQoL with other therapy methods, it was noted that there was a very limited number of studies that considered the effect of SDF on the OHRqoL [19]. Although it was found that the OHRQoL was not notably affected by SDF when compared to other treatment options, this conclusion was constrained by the small number of studies in this field.

The purpose of this study is thus to evaluate the impact of SDF treatment on the OHRQoL of uncooperative children aged 0–5 years three months after treatment using the ECOHIS-G. We hypothesized that the impact of pain relief on the OHRQoL outweighs the negative impact connected to the aesthetic impairment of teeth treated with SDF. This study thereby aims to add to the sparse body of research, especially as there have been no studies in Austria so far on the effects of SDF on the OHRQoL of preschool children, closing an important lacuna in the scientific landscape.

## 2. Materials and Methods

### 2.1. Study Design, Subjects, and Setting

For this prospective pilot study, patients aged 0–5 years were recruited from the Department for Paediatric Dentistry of the University Clinic of Dentistry in Vienna, Austria between January 2020 and November 2021. The clinical examination was performed by a trained and calibrated examiner (SA) according to the dmft index and WHO criteria [20] using artificial light, an air/water syringe, a standardized dental probe, and dental mirror. Calibration was performed using standardized methods and clinical pictures for the assessment of dental caries. The lesion activity was explored with a CPI probe, and every surface of the carious tooth was examined. An active lesion was present if softness was detected upon gentle probing, whereas the surface of inactive caries was hard to probe. The plaque index was assessed using the simplified additive index for plaque accumulation described by Ambjornsen et al. [21] (score “0” = no visible plaque, score “1” = visible plaque).

Only children with compliance and behavioral problems aged 0 to 5 years old with at least one carious deciduous tooth without symptoms of pulpal involvement were included. The included teeth had either occlusal and/or buccal active caries lesions, approximal lesions were excluded. If a child was allergic to silver, iodine, or had any hereditary dental alterations of teeth, they were not eligible for participation, as well as children with severe medical problems or emergent dental needs. After clinical examination, parents were given oral hygiene and diet recommendations. They were instructed to brush their child’s teeth twice a day with a fluoridated toothpaste.

The main components of SDF are water, silver (255,000 ppm), ammonia, and fluoride (44,800 ppm). The application of 38% SDF (Riva Star, SDI, Bayswater, Australia) on the carious lesions was followed according to the protocol provided by the manufacturer: (1) teeth were cleaned with prophy paste, and the area was isolated with cotton rolls; (2) lips were protected with cocoa butter; (3) the solution was carefully applied from the silver capsule to the treatment site only; (4) a generous amount of solution from the green capsule was applied to the treatment site until the creamy white turned clear; (5) teeth were blotted dry; and (6) the patient’s caregiver was instructed that the child should not to drink or eat for the next 30 min.

The enrolment in this study was voluntary. The legal guardian of the patient was informed about the study verbally and signed an informed consent form after verbal assent was obtained. This study was approved by the ethics committee of the Medical University of Vienna (AZ: 1822/2015).

### 2.2. Variables

To assess the child’s OHRQoL before and after treatment, the German version of the ECOHIS (ECOHIS-G) was used. The questionnaire consists of 13 questions and is divided into two main parts, namely, the child impact section (9 items) and the family impact section (4 items). The child impact section contains four subscales: symptom (one item), child-related function (four items), psychology (two items), and self-image (two items). The family impact section comprises two subscales: parental distress (two items) and family-related function (two items). Questions ask about the frequency of events in the child’s entire life. Responses are made on an ordinal scale (0 = never, 1 = hardly ever, 2 = occasionally, 3 = often, 4 = very often). An additional answer option “do not know” exists in every item, which was treated in the analysis as a missing answer. Summing the response codes for the questionnaire items generates domain scores and an overall ECOHIS score.

Thus, the highest scores result in a less favorable OHRQoL. Only parents who had full command of the German language and could complete the ECOHIS-G without any help were included in this study.

### 2.3. Data Sources and Measurements

The dentist and dental assistants informed all participants about the format of the present study. If a patient fulfilled all inclusion criteria, the participant and caregiver were asked about any prior diseases in the previous month that could influence the OHRQoL. After ruling out any exclusion criterion, the questionnaires were distributed after written consent from the children’s caregiver was obtained. The ECOHIS-G was filled out at baseline (T0) and three months (T1) after therapy. Questionnaires with more than two missing items were excluded from the study and further analysis. To ensure the patient’s anonymity, every participant´s name was replaced with a randomly assigned number.

### 2.4. Statistical Methods

Statistical analysis was performed using the IBM SPSS Statistics software (SPSS version 27; Chicago, IL, USA). The following variables were investigated: age (in years), sex (male/female), dmft/DMFT index, and plaque accumulation. Only deciduous teeth with caries lesions without pulpal involvement were included in this study.

Data analysis included descriptive statistics and the Wilcoxon signed-rank test to determine the significance of differences in the overall ECOHIS scores before and after therapy in each group. The scores of each domain (oral symptoms, functional limitations, emotional wellbeing, and social wellbeing) of the ECOHIS-G were added, and a mean value and range were calculated. *p*-values and 95% confidence intervals (CI) were calculated and considered significant if less than 0.05. The effect size was calculated to reveal the strength of the statistical change. For all other mean difference tests for variables such as sex and age, the same criteria for significance were applied.

## 3. Results

All participants completed the ECOHIS-G questionnaire before treatment and three months after SDF application. Table 1 presents the demographic and clinical characteristics of participants. A total of thirty children (2.8 [±1.0] years) aged 0–5 years with 79 active carious lesions, which required treatment with SDF, were enrolled in the study (Table 1). There was an even distribution of females and males (14:16).

At baseline, the mean dmft for children who completed the followup (*n* = 30) was 4.2 (±2.9). The mean (SD) number of SDF-treated teeth was 2.6 (±1.3). About 41% (*n* = 32) of the treated lesions were in posterior teeth and 59% (*n* = 47) in anterior teeth. After three months, all (100%) participants remained in the study. However, two participants were excluded from the analysis as more than two items (out of 13 items) in the ECOHIS questionnaire were missing.

In Table 2, the ECOHIS results before and after therapy are listed. The overall ECOHIS score (21.4 [±8.5] to 16.3 [±5.6]) and child impact section scores (14.9 [±6.7] to 10.8 [±5.2]) decreased significantly (*p* < 0.05), demonstrating moderate to high effect sizes (Table 2). The domain “child function” (5.8 [±2.4] to 3.9 [±2.0]) and “child symptoms” (2.9 [±2.1] to 2.0 [±1.3]) presented the greatest decrease in the ECOHIS scores. The effect size for almost all domains in the child impact section were large, except for the social interaction section (0.2), which was small. The largest effect size was observed in the section “child function” (1.00), followed by child symptoms (0.7). The ECOHIS scores for the domains “parent distress” (0.1) and “family impact section” (0.2) showed the smallest effect size and lowest change. The *p*-value for the child impact section and domains “child symptoms” and “child function”, as well as the ECOHIS total score were lower than 0.05 suggesting that the changes were significantly different for that level of significance. The overall OHRQoL of the children significantly improved following the 38% SDF protocol.

**Figure 1:**
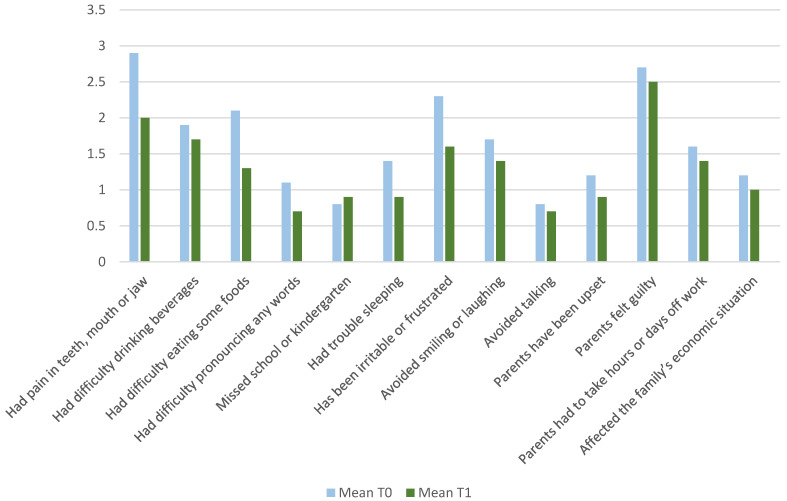
Responses to the German Early Childhood Oral Health Impact Scale (ECOHIS-G) items.

The mean scores of each ECOHIS item at baseline and followup examination are displayed in Figure 1. At baseline, the items “had pain in the teeth, mouth or jaw” (2.9 [±1.6]), “has been irritable or frustrated” (2.3 [±1.1]), and “had difficulties eating” (2.1 [±0.9]) were the most frequently reported items in the child impact section. After SDF treatment, the item “feeling guilty” (2.7 [±0.6] to 2.5 [±0.9]) in the family impact section was almost similar compared with the value reported at the baseline examination.

## 4. Discussion

In the past 10 years, there has been an increased use of general anesthesia and sedation for pediatric dental treatments often linked with multiple dental caries [22]. At the same time, due to the risks associated with general anesthesia in small children, there has also been an observable shift in treatment of children who are not compliant under DGA towards the use of the caries inhibiting liquid SDF. We hypothesized that SDF has a positive overall impact on the patients’ OHRQoL. Our overall results support our hypothesis. They showed a significant overall improvement in the OHRQoL after treatment of teeth with SDF.

Ruff et al. conducted a meta-analysis comparing the influence of SDF on the OHRQoL versus other standard therapy methods [19]. The findings showed that the improvement in OHRQoL after treatment with SDF did not differ from results with other treatment options such as atraumatic restorations. This corroborates findings of recent studies conducted in Australia, Saudi Arabia, and China [23,24,25] that reported similar findings regarding an improvement in the ECOHIS sum score. For example, Yawary et al. [23] showed that dental caries management with a single application of 38% SDF and comprehensive oral health education had a similar ECOHIS total sum at baseline (20.9 [±10.5]) and after therapy (16.7 [±9.3]) as our findings.

In the child impact section, we observed that the domains “child symptoms” and “child function” were significantly (*p* < 0.05) lower after therapy than at baseline.

This concurs with recent studies from Renugalakshmi et al. [24], as the lowest ECOHIS scores were also found in the same domains, which accords with our results indicating that caries lesions compromise functionality of chewing and drinking due to hypersensitivity and or pain. The use of SDF on carious lesions led to a significant decrease (*p* > 0.05) in both studies in this domain showing that an effect was already detectable after a few months.

What stands out in our findings is that the overall average increase in the child impact section was greater than the score of the family impact section, which almost remained the same. Surprisingly the strongest increase was seen in the item “feeling guilty”. This might be stronger for carious lesions treated with SDF than for other dental treatments that do not have aesthetic limitations, as these teeth present a more likely reminder to the caregivers of failed oral hygiene, which is also publicly visible. Before treatment with SDF, caregivers are thoroughly instructed about the side effects and therapy success; therefore, parents, who are unhappy with the aesthetic consequences, choose to decline treatment or opt for a solution that involves sedation or an alternative. This means that those participating clearly put more weight on their child’s dental functioning than those that do not opt for SDF. This aligns with a recent study from Duangthip et al. [25] who demonstrated that this item also had a higher value (66.4%) after treatment than at the baseline examination. The authors also interpreted this fact as an expression of the parent’s twinges of conscience due to their inability of providing their child with sufficient oral hygiene leading to pain and aesthetic compromises. The nonsignificant scores in the item “avoided smiling or laughing” might not be as pronounced as in the domains that comprised our participants, due to the age group of the participants, as these children did not yet attend kindergarten or school. Since social interactions have a larger impact on the ECOHIS that concern aesthetics aspects, this might explain the insignificant change observed in these sections.

The limitations of our study partly arose from the use of the ECOHIS, which is a parental proxy measure to assess a child’s wellbeing. This might be a weak point insofar as parents miss certain behavioral cues or are biased due to misinterpretation or insufficient recall of symptoms and pain. Since half of the children (56%) were around the age of two or three, parents must rely more on indirect cues, than in cases where the child is four or older, as they are better capable of articulating dental problems. Another potential limitation was the 3-month study period. There are several short-term clinical studies that show promising caries arrest results in children [24,26]. However, further followup examinations are planned at 6 and 12 months after the first application of SDF.

Another limitation was the small sample size, which is why this study is a pilot study, which makes salient points for which followup is required. A larger number of patients might allow us to draw more nuanced conclusions regarding the tradeoff between parental pangs of conscience and benefits from freedom of pain. The complexity and difficulty of recruiting a much larger sample size should be highlighted. We only collected participants aged 0–5 with behavioral management problems who were not able to be treated with different or alternative methods. Furthermore, we carefully selected patients with specific carious lesions without any symptomatic pulpal involvement, tooth mobility, and genetic dental defects, which made the inclusion highly selective. This should be kept in mind when planning a similar study with a larger sample size.

## 5. Conclusions

We observed a significant improvement in the OHRQoL in children aged 0–5 years who were treated with SDF due to behavioral problems. It is a promising treatment method for uncooperative children and an efficient way to bridge the time gap until a conventional restorative treatment is possible.

## Figures and Tables

**Table 1:** Summary of Sample Data (*n* = 30 patients).

	All *n* (%)
Sex	
Male ♂	16 (53)
Female ♀	14 (47)
Age	
Mean (SD)	2.8 years (1.0)
0–1 years	6 (20)
2 years	10 (33)
3 years	7 (23)
4 years	4 (13)
5 years	3 (10)
Baseline	
dmft index *, mean (SD)	4.2 (2.9)
Plaque	36 (17.2)
Distribution of teeth	
Mean (SD)	2.6 (1.3)
Molars	32 (41)
Incisors	47 (59)

* dmft = decayed, missing, filled tooth.

**Table 2:** Overall Early Childhood Oral Health Impact Scale (ECOHIS), child impact section (CIS), and family impact section (FIS) scores (Baseline, T1) for children who received SDF treatment.

ECOHIS Domain	Baseline (T0) Mean (SD)	Followup (T1) Mean (SD)	Effect Size	*p*-Value
Total Score	21.4 (8.5)	16.3 (5.6)	0.7	<0.001
Child impact	14.9 (6.7)	10.8 (5.2)	0.6	<0.001
Symptoms	2.9 (2.1)	2.0 (1.3)	0.7	<0.001
Function	5.8 (2.4)	3.9 (2.0)	1.0	<0.001
Child Psychology	3.7 (1.9)	2.9 (1.6)	0.4	0.62
Social interaction	2.5 (2.2)	2.1 (1.8)	0.2	0.48
Family impact	6.7 (3.9)	5.8 (4.1)	0.2	0.09
Parent distress	3.9 (2.2)	3.5 (2.4)	0.1	0.34
Family function	2.8 (1.4)	2.5 (0.9)	0.2	0.11

## Data Availability

The datasets of this article are available from the corresponding author on a reasonable request.
